# Bringing Code to Data: Do Not Forget Governance

**DOI:** 10.2196/18087

**Published:** 2020-07-28

**Authors:** Christine Suver, Adrian Thorogood, Megan Doerr, John Wilbanks, Bartha Knoppers

**Affiliations:** 1 Sage Bionetworks Seattle, WA United States; 2 Centre of Genomics and Policy McGill University Montreal, QC Canada

**Keywords:** data management, privacy, ethics, research, data science, machine learning

## Abstract

Developing or independently evaluating algorithms in biomedical research is difficult because of restrictions on access to clinical data. Access is restricted because of privacy concerns, the proprietary treatment of data by institutions (fueled in part by the cost of data hosting, curation, and distribution), concerns over misuse, and the complexities of applicable regulatory frameworks. The use of cloud technology and services can address many of the barriers to data sharing. For example, researchers can access data in high performance, secure, and auditable cloud computing environments without the need for copying or downloading. An alternative path to accessing data sets requiring additional protection is the model-to-data approach. In model-to-data, researchers submit algorithms to run on secure data sets that remain hidden. Model-to-data is designed to enhance security and local control while enabling communities of researchers to generate new knowledge from sequestered data. Model-to-data has not yet been widely implemented, but pilots have demonstrated its utility when technical or legal constraints preclude other methods of sharing. We argue that model-to-data can make a valuable addition to our data sharing arsenal, with 2 caveats. First, model-to-data should only be adopted where necessary to supplement rather than replace existing data-sharing approaches given that it requires significant resource commitments from data stewards and limits scientific freedom, reproducibility, and scalability. Second, although model-to-data reduces concerns over data privacy and loss of local control when sharing clinical data, it is not an ethical panacea. Data stewards will remain hesitant to adopt model-to-data approaches without guidance on how to do so responsibly. To address this gap, we explored how commitments to open science, reproducibility, security, respect for data subjects, and research ethics oversight must be re-evaluated in a model-to-data context.

## Introduction

Sharing health data is essential to accelerate knowledge and discovery through opportunities for replication, validation, meta-analysis, and creative reuse [1]. Indeed, many research funding agencies, institutions, and scientific journals encourage or even require the disclosure of research data to the broader scientific community as a means to foster collaborations and to increase scientific accountability, transparency, and reproducibility [2]. Traditionally, data generated as part of research projects or routine clinical care are shared with the scientific community by means of direct download, with the data recipients analyzing data in their local computing environments. However, it is impractical to share some data sets in this manner because of their large size or because of legal restrictions on transfer between institutions or across sovereign borders. One common category of legal restriction is the confidentiality requirement applicable to health information, such as those required in the United States by the *Health Insurance Portability and Accountability Act* [3]. Another category of legal restriction is the limitations on cross-border transfers of certain data. For example, the European Union restricts the transfer of personal data to external countries under its *General Data Protection Regulation* *2016/679* (GDPR) [4]. Finland’s *Secondary Use Act* requires health care data, including nonidentifiable data, to be processed in a secure data center based in Finland [5]. For more examples internationally, see International Compilation of Human Research Standards [6]. Furthermore, researchers and health care institutions who generate data may be concerned over relinquishing data assets given their perceived commercial or academic value, the costs of organizing and annotating data, the confidentiality and security of individual-level data, and loss of oversight of future data use.

The traditional data sharing governance role of health care institutions has been as custodians of data, whose primary responsibility was to keep data secure and confidential. This was achieved through silos, as illustrated by the patchwork of distinct health and accounting records one accumulates when navigating health care systems from hospitals to specialty clinics. The move to open data governance for broadening data sharing practices since 2000 represents a shift of that philosophy, one in which the community of data recipients is collectively responsible for protecting and maintaining the integrity of the data [7]. Open data is attractive in some ways, precisely because it externalizes the very real financial and logistical costs of data governance while providing opportunities for new research perspectives. However, open data governance may be insufficient for many holders of clinical data sets with individual-level privacy or intellectual property concerns.

In between data custodianship and open data governance, data stewardship is an institutional commitment to maximizing the organizational, scientific, and societal benefits of data sharing when also protecting data against privacy and security breaches and misuse [8]. Data stewards may be data generators themselves (eg, hospitals or research institutions) or an honest broker who acts as an independent mediator and trusted partner on behalf of one or more data generators and data users. An honest broker is generally mandated contractually or otherwise to protect and manage secure access to data under the ultimate legal control of another organization.

Data stewards (whether data generators or honest brokers) can now adopt different technical models for making data accessible to users. Typically, a data steward provides access by transferring copies of data to users. This copy-and-download model, however, raises concerns about unaccountable data management and use. Two alternative models promise greater security. In a researcher-to-data approach, a steward makes data available to users within a secure and auditable (cloud) computing environment. In a model-to-data approach, users can submit queries or algorithms to run on secure, hidden data. Each data access model involves different divisions of costs between stewards and users as well as tradeoffs between data use and data protection.

The first part of our paper compares these models primarily in the context of a single health data resource, controlled by a single entity (although this could be a pooled or centralized resource). We highlight the challenges that arise when attempting to scale each data access model beyond single health data resources to networks of multiple resources. The second part of our article focuses on the model-to-data approach. Admittedly, there are only a few existing implementations of model-to-data, and all these essentially involve single resources. Scaling model-to-data to connect multiple resources requires the establishment of a federated data system and, at this time, these systems remain to be largely theoretical. We argued that model-to-data can be a valuable addition to our data sharing arsenal, but it should only be adopted where truly necessary. This approach should supplement rather than replace existing data sharing approaches for the following reasons. First, model-to-data tends to limit scientific freedom and reproducibility. Second, although model-to-data reduces concerns over data privacy and loss of local control when sharing clinical data, it is not an ethical panacea. Model-to-data approaches require just as much, if not more, attention to matters of ethical and legal governance as other data sharing approaches. Third, furnishing the appropriate infrastructure and expertise for a model-to-data approach requires a significant and sustained investment of resources. Misaligned incentives remain to be a major barrier to data sharing, generally [1]. The challenges of aligning incentives are likely to be exacerbated for model-to-data, given the associated expenses of hosting both data and analyses. Finally, all the aforementioned scientific, legal-ethical, and resource challenges also threaten the scalability of model-to-data from single data resources to networks of multiple resources (federated data systems).

## Technical Data Access Models

**Figure 1:**
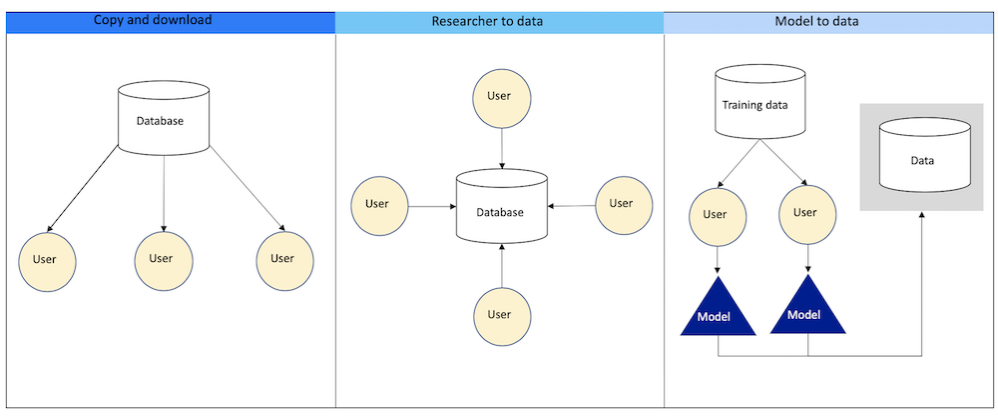
Common Data Sharing Models.

Data sharing has long been synonymous with a copy-and-download approach, where data stewards transfer copies of data sets to researchers. The resulting loss of control over data raises a range of privacy, credit, and proprietary concerns. Access governance mechanisms, such as due diligence review of access requests by a data access committee and data access agreements, can mitigate these concerns to some degree, but may limit access by researchers who do not have a recognized institutional affiliation. Data recipients must also have the expertise and means to provide data hosting, management, and analysis environment. Emerging technical data sharing models aim to further alleviate tradeoffs between data use and data protection (Figure 1). In a researcher-to-data approach, instead of transferring a copy of the data to data recipients, researchers programmatically access data sets that are maintained in a secure computing environment. This approach arose from the development of cloud computing. Cloud cyberinfrastructure has successfully expanded programmatic access to data and analysis tools to a global community of researchers, in many cases cheaply and efficiently, without the cost and logistical challenges of data transfer [9]. These secure computing environments are sometimes characterized as virtual data enclaves or data safe havens [10,11]. A data safe haven implies a collective resource kept in a secure computing environment and managed with appropriate ethical and legal governance for the mutual benefit of individuals, communities, and the society. Data safe havens can enable investigators to conduct exploratory research or can limit investigators to conduct a preapproved analysis, but only results, not individual data, are permitted to leave [11]. An example is the Centers for Medicare & Medicaid Services Virtual Research Data Center, which allows research on Medicare and some Medicaid claims [12].

Data safe havens address the costs, risks, and logistical challenges of sharing large, sensitive data sets by obviating the need for copying and distributing the data. Additionally, the cloud platform may allow researchers without intensive local computing resources to run complex analytical models in rented cloud computing infrastructures. Nonetheless, data safe havens do not fully resolve concerns over privacy leaks and misuse because data users must still be trusted to some degree to keep the data within the secure environment and to use the data appropriately. However, these risks can be reduced through access oversight, data use agreements, and active monitoring. Finally, because users can directly interact with data in secure environments, data stewards can share at least some of the costs of data hosting and curation with them.

A comparison of the technical data access models has been provided in Table 1.

A more secure approach has been piloted to allow researchers to extract knowledge from sensitive data sets that remain sequestered. In a model-to-data approach, researchers submit their analytic code or model to a data steward, who maintains the restricted data set in a secure computing environment. The data steward runs the code and returns outputs (eg, summary or performance statistics) to researchers. Data are not moved or even made directly accessible to data users. Model-to-data is made possible by container technologies (eg, Docker) that simplify the bundling and transfer of software models and their dependencies across computing platforms [13]. The utility of model-to-data has been successfully demonstrated in crowdsourced research competitions involving confidential medical data [14,15]. For example, the Digital Mammography DREAM Challenge enabled the analysis of >640,000 deidentified digital mammography images from >86,000 individuals, without transferring or providing direct access to the images [13]. Lisa Austin and David Lie incorporate a version of model-to-data as part of a safe sharing sites proposal, where “computations may be performed on the data in a secure and privacy-protective manner without releasing the raw data, and all data sharing is transparent and auditable” [16]. It may be important depending on the context that the safe sharing site is an independent, honest broker. More recently, the Korean OpenData4Covid19 initiative used a model-to-data approach to share novel coronavirus disease–related health care data with researchers worldwide to fight the pandemic. Researchers must submit their analyses to be run by the Korean Data Centre. Only aggregate results are returned [17]. This example demonstrates how model-to-data can allow data to be rapidly shared with a broad community of users than would otherwise be possible.

We discussed the 4 categories of challenges confronting the model-to-data technical data access approach: (1) scalability to multiple resources, (2) scientific governance, (3) legal-ethical governance, and (4) incentives and sustainability. Data stewards must consider each of these challenges to determine if and how a model-to-data approach should be adopted in a particular context.

**Table 1:** Comparison of technical data access models.

Characteristics	Copy-and-download	Researcher-to-data	Model-to-data
Costs of data curation	Shared between data steward and data user	Primarily borne by data steward	Completely borne by data steward
Cost of computing infrastructure	Borne by data user	Borne by data steward (users may be charged for compute)	Borne by data steward (users may be charged for compute)
Researcher freedom (eg, methods and tools)	Greatest degree of freedom. Limited by terms of use	Limited freedom. Limited by computing infrastructure and terms of use	Least freedom. Limited by API^a^ structure and computing infrastructure
Security and confidentiality of *individual-level data*	Weak. Many copies of data shared with many users, possibly in many countries, subject only to data access agreements. Difficult to audit. Difficult to withdraw data once shared	Strong. Data shared with third party users within a secure and auditable computing environment. Data can be easily withdrawn	Very strong. Data remains hidden from users. Data can be withdrawn at any time
Data privacy protection	Weak. Deidentification and data access agreement limiting reidentification	Strong. Individual-level data may be viewable but not downloadable. Only results are released. Outputs may need to be deidentified	Very strong. Individual-level data are not downloadable or viewable. Only results are released
Security and confidentiality of *Researchers’ Ideas or Code*	Very strong. Researchers submit proposals but maintain control over methods	Medium. Researchers’ activities are supervised/audited	Weak. Researchers must share query/workflow with data steward
Informed consent	Consent may be needed for research use, sharing, and cross-border transfer	Consent may be needed for research use only	Consent may be needed for research use only
Research ethics oversight	Data user may need research ethics approval	Data steward may need to provide research ethics approval	Data steward may need to provide research ethics approval
Scalability to multiple resources	Straightforward through a distributed data commons, with some shared infrastructure (eg, access portal)	Can only be done indirectly through an individual-level meta-analysis	Difficult but theoretically possible through a federated data system
Legal agreements	Data access/transfer agreement and data use agreement	Data use agreement and computing environment terms of use	Data use agreement only

^a^API: application programming interface.

## Scalability of Model-to-Data to a Network of Multiple Resources

Thus far, we have only considered the case of providing access to a single data resource held by a single data steward. Much of the value of data sharing occurs, however, where multiple data resources are connected into a network. Researchers often seek to achieve greater statistical power or refine an algorithm by aggregating multiple data sets. This is particularly relevant to research requiring a comprehensive analysis of data sets at scale. For instance, efforts in achieving precision medicine rely on the ability to combine a vast amount of diverse data types to investigate health and wellness [18]. There is also a strong interest in combining data across sectors to improve knowledge. For example, AllIn partners are looking for solutions to intersect data from diverse community resources to improve community health outcomes [19]. However, combining data from diverse sources is challenging because of the lack of interoperable standards between various data models (eg, OMOP, PCORnet, and TrinetX) [20].

In addition, data sharing networks or consortia must agree on a shared technical solution, raising additional complexity [21]. Consortia of data stewards can agree to centralize or pool their data into a single repository governed by common ethical and legal governance rules and processes (eg, the *All of Us* Research Program [22]). Once the data are centralized, the data stewards must still decide on a technical data sharing model (ie, copy-and-download, researcher-to-data, or model-to-data). Where data distribution is problematic, a researcher-to-data approach may naturally align with a centralized consortium model. The aggregated data can be hosted in a single, secure computing environment. The stewards of the repository can then grant qualified researchers access to data and tools within the secure environment. Admittedly, centralization still involves relinquishing local control and trusting a third party to govern the data. However, these concerns can still be mitigated by adopting a researcher-to-data or a model-to-data approach as copies of data would not be further distributed to researchers.

Where data cannot be shared or centralized, model-to-data can still theoretically enable a virtual form of data aggregation through a federated data system (ie, distributed data system). A federated data system is composed of a network of autonomous data repositories or nodes that share a common data structure/schema and governance principles, but the data remains localized. Users can run an identical query or analysis at each data repository [23]. The results of the queries or analyses are aggregated and returned to the researcher who submitted the query. A federated data system enabling linkage and analysis of sensitive health care data in multiple repositories is proposed by the European Personal Health Train, where *analytical tasks visit data sources* [24]. Data sets are likened to *stations*, algorithms are likened to the payload delivered by a *train*, and the network is likened to the train *track*. However, because of the variety of challenges explored below, federated data systems remain to be largely theoretical at this time.

Federated data systems potentially provide high levels of local security, control, and sovereignty as data remain hidden at each node in the network. They also potentially allow for the scalability of some analyses across multiple nodes into large, virtual cohorts. In addition, federated data systems can share ethical and legal governance standards (eg, access and use policies) and associated technical infrastructure like user authentication and authorization, such as the researcher passports infrastructure proposed by the Global Alliance for Genomics and Health [25]. Federated data systems can, in theory, also allow for virtual analysis of international cohorts without sacrificing compliance with data localization requirements, such as the cross-border transfer restrictions imposed by the European GDPR [26,27]. With model-to-data, only the results of algorithmic queries, and not the underlying individual-level data, need to leave a particular territory.

Challenges hampering the implementation of federated data systems include reaching and maintaining “agreement and commitment from federation members on the shared goals, operating principles, metrics for success and apportionment of the benefits and commercial value” [23]. Negotiation is also needed to determine what aspects of the data sharing architecture will be common versus what parts will be decentralized, and who will pay for building and maintaining the common aspects. Given that finding consensus solutions about these aspects in large groups can be difficult, large federated data systems may need to appoint a governing body to facilitate decisions among the federated members and designate data stewards at each node to implement the federation’s resolutions. In a federated data system, each node is accountable to all other nodes. Thus, the effectiveness of governance may be inversely proportional to the number of nodes. In short, although complicated, large virtual cohorts can still theoretically be achieved through federated analyses, where algorithms run across multiple secure databases, with aggregation of results.

Many projects are piloting this federated data system approach. For example, the World Economic Forum aims to connect genomic databases from Canada, Australia, the United States, and the United Kingdom to enable queries of rare disease patients. Two European-funded Horizon 2020 projects implementing the federated data system approach include (1) the Research on European Children study and the Adults Born Preterm study, which connects multiple European cohort studies and aims to improve health, development, and quality of life for preterm and low-birth weight individuals [28,29] and (2) EUCAN-Connect, which aims to connect genomic databases to advance precision medicine [30].

Although an attractive alternative to the copy-and-download and researcher-to-data approaches, model-to-data, including federated model-to-data, highlights key scientific, ethical, and legal governance issues that must be addressed as we balance scientific innovation and discovery with data protection.

The current lack of guidance may discourage the real-world implementation of model-to-data solutions. We aim to address this gap by identifying key points to consider for data stewards, focusing on a single data resource model-to-data approach. We conclude that model-to-data should only be adopted in limited circumstances, as a complement to rather than as a substitute for existing data sharing models. This argument applies by extension to the more complicated case of federated data systems involving a network of multiple resources made available through model-to-data.

## Scientific Governance of Model-to-Data

The effectiveness of any data sharing model is a function of the type, quality, and richness of the data; the number of problem solvers who can access the data; the diversity of questions and analysis methods that can be applied; and the ability to validate and compare outputs. Modern data sharing governance philosophy has been popularly articulated according to the *FAIR guiding principles* that data be findable, accessible, interoperable, and reusable [31]. The FAIRness of data is always heavily dependent on the actions and abilities of the data steward. FAIRness is all the more dependent on stewards in the case of model-to-data as the data user has no ability to curate or conduct quality assurance on the data. In this paper, we focus on the case of a single data resource. Many of these issues will be challenging in the case of a federated data system involving multiple resources, as participating resources will all have to agree on standards and cost-sharing arrangements.

### Findable

To be findable, data resources must be associated with rich, accurate, and standardized metadata. Metadata must generally be openly accessible for researchers to discover relevant data sets through data search engines. New technologies such as genomic Beacons and clinical Beacons allow researchers to search for individual-level traits, outcomes, or genetic variants without revealing detailed individual-level data [32]. Beacon technology works through an application programming interface (API), allowing researchers to query a data set (“do you have any patients with genetic variant ABC?”) and receive a simple *Yes* or *No* in response (or some slightly more detailed demographic or clinical information about the individual). Individual-level search may be equally possible across different data sharing models, with appropriate protections in place to ensure that searches do not leak excessive information about individuals. In model-to-data, enabling meaningful discovery may also call on data stewards to provide sufficient metadata or, better, an unbiased, representative training (testbed or sandbox) data set to help users understand the characteristics of the sequestered data set or to develop, train, and test their workflows and machine learning algorithms. The selection, composition, curation, and annotation of the representative training data sets is critical to the success of model-to-data as the training data set is used to *fit* models. Curation of the training (visible) and validation (sequestered) data sets must match perfectly for the data to be truly *findable*.

### Accessible

Model-to-data enables analyses of sequestered data that cannot be directly accessed by researchers. At least superficially, the model-to-data approach is one way to resolve tensions between openness and confidentiality. In model-to-data, the data steward does not focus on securely transferring data to trustworthy and qualified users or on controlling access to a cloud computing environment, yet the data steward may still play an important governance and scientific oversight role. Determinations may still need to be made about who is authorized to analyze the data (ie, who can submit code to run on the data), and these determinations would need to be enforced through defined user authentication and authorization processes. In this manner, the model-to-data data steward will need to play a role akin to traditional data access committees in the review of data user access requests. Data stewards may also need to take on a more hands-on scientific advisory role, reviewing the appropriateness and scientific validity of the submitted analysis codes, providing feedback on model performance, and troubleshooting. Further complicating the responsibilities of the model-to-data data steward is the reality of finite computing and advisory resources. Because data are not directly accessible, model-to-data may enable data stewards to expand access to a wider group of data users than would otherwise be allowed because of reduced concerns over security and privacy. However, broadening access would need to be balanced with the need to be parsimonious against limited resources.

### Interoperable

The responsibility for making data interoperable in model-to-data rests primarily with the data stewards, as data users cannot access the data directly. Furthermore, data stewards must ensure that their computing environment is interoperable to enable the third-party code to run accurately. This need for interoperable platforms is encouraging harmonization of not only data collection approaches but also technology architectures, data ontologies, formats, and governance structures. Indeed, the Global Alliance for Genomics and Health [33] has proposed a federated international ecosystem of genomic databases for which it is developing technical standards and policy frames to make the data available to authorized data users under relevant conditions [34]. The GA4GH Cloud Work Stream developed standardized computing routines, protocols, and tools (APIs) to enable the portability of algorithms, tools, and workflows across cloud environments in large-scale distributed projects [35]. These standards are being piloted in GA4GH Driver Projects, including the National Cancer Institute Genomic Data Commons [33,36] and the Canadian Distributed Infrastructure for Genomics [37]. These ongoing efforts to harmonize data ontologies, formats, and workflows support the interoperability of data sets both in the cloud as well as in the model-to-data research.

### Reusable

The goal of data reuse is to enable the generation of new knowledge from data and to enable validation of previous findings to promote scientific consensus. In order for data to be reusable through model-to-data, data stewards must ensure that the data are high quality, rich, and fit-for-purpose. Preparing data for analysis is often the most taxing part of data science [38]. Regardless of the data sharing model, data generators and stewards bear a significant degree of responsibility for data curation. There are also efforts to harmonize clinical assays as evidenced by the proposed *Diagnostic Accuracy and Innovation Act* [39]. Where copies of data are distributed, researchers can participate to various degrees in curating data to render it usable for specific purposes. In model-to-data, however, the responsibility for curation and annotation of the sequestered data set is entirely the responsibility of data stewards.

### Ensuring the Quality of Research Outputs

A concern for data sharing generally is not only ensuring the quality of data but also the quality of research outputs. These results are usually in the form of summary statistics, but increasingly data science outputs also include trained algorithms. Usually, the quality of research outputs is left to quality control by journals and peer review. Owing to their intensive role in data curation and algorithmic facilitation, in some cases, model-to-data data stewards may insist on coauthorship or may reserve the right to review abstracts or manuscripts before publication, although these approaches can raise concerns about scientific freedom.

## Ethical and Legal Governance of Model-to-Data

Considerations for ethical data sharing go beyond scientific concerns. Data stewards adopting a model-to-data approach must also address issues of privacy, confidentiality, security, ownership, research ethics oversight, and sustainability.

### Privacy, Confidentiality, and Security of Patients’ Data

Concerns about risks to privacy are at the core of resistance to data sharing. Data sharing models involving the distribution of data to researchers rely heavily on deidentification (the process of removing direct identifiers). However, the residual reidentification risk of even limited genetic or demographic data sets has been clearly demonstrated [40]. Data access governance mechanisms (eg, due diligence review of access requestors, data access agreements) may mitigate risks, but ultimately depend on trust [41]. There are few means available to monitor obligations to keep data confidential and secure and, even if a breach is detected, it is not clear if and how a breach could be sanctioned. In model-to-data, by contrast, no copies of data are distributed, reducing privacy risks. Data stewards do not have to predict all possible future reidentification scenarios or assess the trustworthiness of data requesters to keep data secure when they enable model-to-data research.

However, there remain some limited privacy concerns regarding model-to-data. First, to make model-to-data data sets usable, limited training sets of data may need to be distributed. It may be possible to distribute synthetic or *noisy* training data sets as a way to preserve the data subject’s privacy and data confidentiality. Second, privacy risks may differ depending on whether model-to-data access is provided for algorithm training, validation, or application. Unintended memorization of data has been shown to occur during the training of algorithms [42]. Outputs from applying a model could contain identifiable information, or such information may be reverse engineered or inferred. Privacy leaks can be reduced by adding safeguards such as auditing of outputs and limiting what kinds of outputs may be returned to researchers. Even when the release is limited to outputs or knowledge, there may still be a need for careful risk assessment and calibration between openness and privacy protection.

### Informed Consent and Research Ethics Oversight

Whereas model-to-data may allow data stewards to meet data confidentiality and localization requirements, *ethical* concerns remain related to the principles of informed consent and research ethics oversight. Ethically, patients have an interest in knowing who is being provided access to their data and what their data are being used for, even if those parties never directly access their individual-level data. Data privacy laws, particularly the European GDPR [4], require a legal basis, such as consent or public interest, to be able to analyze personal data for certain research purposes. The US National Institutes of Health’s dbGAP genomic data repository imposes ethical data use limitations on data based on the scope of the participant’s consent [43]. With model-to-data, *legal* constraints on use may appear to be diminished or inapplicable as individual-level data are not disclosed to third parties. Nevertheless, data use may raise ethical and social license concerns if data were used by controversial parties or for controversial purposes without patients’ knowledge. Data stewards are responsible for ensuring appropriate oversight of data use. This responsibility overlaps with the scientific oversight mechanisms discussed above.

A traditional mechanism to ensure the ethical conduct of health research is to subject project proposals to approval and oversight by an independent research ethics oversight body (eg, Institutional Review Board, Research Ethics Committee). In a model-to-data context, however, the locus of research ethics oversight may need to shift from the data user’s institution to the data steward, where the actual analysis of data is performed. This shift may in fact allow access to a wider range of data users from outside academic or health care institutions—who would not necessarily be subject to human subject research regulations—to analyze data (eg, commercial researchers and citizen scientists). The data steward would essentially provide a research ethics review as a service to these users. In the case of a federated data system involving multiple data stewards, some level of coordinated research ethics oversight between stewards would need to be developed. For example, a data user could receive approval from one data steward in a network and the other stewards could mutually recognize that approval. Fortunately, models for coordinated oversight of data-intensive research have already been developed and could be adapted to the model-to-data context [41,44].

### Confidentiality and Security of Researchers’ Ideas and Software

A central strength of model-to-data is that it improves data security. However, the trust patterns required to enable security in model-to-data research are novel. In traditional data sharing models, data subjects and data generators must trust data users to secure individual-level data. In model-to-data, by contrast, data users must trust that data stewards will keep their queries and code secure from unauthorized access and that data stewards will not appropriate the ideas or internet protocols of data users for themselves. This issue becomes more problematic when scaling model-to-data to multiple resources. From this perspective, allowing an honest broker to act as the data steward in model-to-data may be desirable (although this would require data generators to transfer control over their sensitive data to the honest broker). Involving an independent trusted third party can reduce real or perceived conflicts of interest. Another potential solution analogous to privacy-preserving record linkage is to enable secure computation without the data steward being able to directly inspect the query or code [45,46]. The query or code is encrypted and sent to a trusted third party to unencrypt at runtime. However, this may be technically complex and data stewards may insist on inspecting the nature of the submitted algorithm. Model-to-data stewards must also consider the security risks associated with allowing outside code to be run inside their protected computing environment (eg, tampering). Providing read-only access to data directories limits the risks that input data may be altered. Preventing network access while executing the analysis code reduces the risk that users may accidentally download data.

## Data Sharing Incentives and Sustainability

The FAIRness of data in model-to-data contexts is heavily dependent on the role of the data steward. The FAIRness of analysis workflows is also increasingly key for the reproducibility of data-intensive health research. In the context of model-to-data, this FAIRness may additionally be essential for trust between data users and data stewards [24,47]. Data stewards need to possess not only *traditional* data governance expertise but also significant computational and analysis skills, a rare combination, and one without formally recognized qualifications at this time. These issues of oversight may hamper the adoption of model-to-data. Ultimately, by shifting more responsibility for data curation and infrastructure costs to data stewards, model-to-data represents a fundamental recalibration of the tripartite responsibilities of data generators, data users, and funders envisaged by the Toronto Statement on Pre-publication Data Sharing, a policy promoting rapid data sharing by large-scale community resource projects in biology [48]. Although model-to-data may seem like a technical decision, it significantly exacerbates the incentive problem already plaguing data sharing [49]. To succeed, model-to-data requires dedicated support from funders as well as from the highest level of leadership within organizations seeking to share data.

Similarly, all databases face sustainability challenges [50]. Who pays for the development, management, and maintenance of data sets, especially over time? For model-to-data, similar questions must also be asked about the sustainability of maintaining computing environments and security infrastructure. Open data distribution models promise a cheap solution to the preservation of data sets in part through the existence of numerous copies of the data set. In model-to-data, a data steward is trusted with preserving the data set and providing access to it over time. Different sustainability models may need to be explored, from fee-for-access to sustained public funding. The choice of model may be influenced by the source of investment in data generation, curation, and infrastructure. Private investors may want to commercialize data access, whereas public funders may insist on an open science approach. One solution could be a commitment by funders to pay for stewardship as a utility contract, one that simultaneously sustains infrastructure and explicitly requires strict platform neutrality. Good contracting practices that guarantee the portability of data sets from steward to steward in the event of bankruptcy or bad faith would also be essential. Furthermore, data stewards must be careful to avoid conflicts of interest toward either the data generators who entrust them with the data or data users who entrust their hypothesis and analysis methods to them. Ultimately, the question for model-to-data approaches and, by extension, federated data systems, is whether the benefits to science and society are worth the cost. The first-of-its-kind economic evaluation of federated data systems concluded that their return on investment was unclear [51].

The major concerns related to sustainability in model-to-data are the evaluation and reproducibility of model-to-data research, the key to research quality, and transparency. If a data steward were to refuse or discontinue algorithmic access services, this would imperil research reproducibility. This issue becomes greater at scale. In a federated data system, all it takes is for one data steward in a network to withdraw algorithmic access to undermine reproducibility. The reproducibility of model-to-data research may be vulnerable to the caprices of funders, scientific priorities, politics, or even individual data subjects. Indeed, because there is only one copy of a data subject’s data in model-to-data, it may also be easier for subjects to withdraw their data. On the one hand, model-to-data supports individual autonomy, allowing individuals to control how their data are (or are not) shared and used over time, which may bolster public trust and willingness to share. On the other hand, this may make reproducibility of research more susceptible to the caprices of individuals.

## Conclusions

Model-to-data promises to unlock health data currently stuck behind institutional (fire)walls and country borders, providing access to researchers without sacrificing security, confidentiality, or local control. Model-to-data can improve the quality of research outputs by providing access to more data.

For algorithms, model-to-data access can serve as a means of rapid, continuous quality control. The accuracy of algorithms can be determined or compared through benchmarking, where the algorithm is run on a standard data set. For traditional research findings, model-to-data may allow peers to reach consensus about insights if running different models generates similar findings. In practice, however, this technical access model involves significant costs and drawbacks. It falls short of its utopian promise of technology, overcoming legal and human constraints on data sharing. We have argued that model-to-data should only be adopted where necessary to supplement rather than to replace existing data sharing approaches for scientific, legal-ethical, and resource reasons. By segregating researchers from data, model-to-data tends to limit scientific freedom, integrity, and reproducibility. Although model-to-data scores high on security and confidentiality, ethical concerns persist about individual control over who uses their data and for what purposes. Model-to-data also presents a coordination puzzle for research ethics oversight systems, with the research analysis occurring in an institution different from that of the researcher.

Model-to-data intensifies the incentive issues already plaguing open science [49]. Data stewards must provide expertise and resources to curate data, maintain technical infrastructure, and conduct scientific activities on behalf of users. Meanwhile, direct benefits tend to accrue to data users. Scalability is another concern for model-to-data. Scalability from a single resource to multiple resources is possible with a federated data system. However, as these systems scale, the challenges of maintaining scientific data harmonization, technical standardization, and agreements about cost and benefit sharing scale as well. The scale of federated data systems may therefore be naturally limited to a smaller number of trusted parties.

We predict that the model-to-data approach will become an increasingly attractive option for particular use cases as the scale, diversity, and regulatory complexity of international data sharing continue to rapidly increase. This assumes that the scientific, legal-ethical, incentives, and network issues described above are addressed. Model-to-data will be most useful for large-scale stand-alone resources or small networks of resources when scientific data standards are well established, where the inquiry is clearly defined in advance (eg, in a hackathon challenge), and when providing access to a specific community of users who would not otherwise be authorized. Ultimately, the success of model-to-data may require the migration of expertise in data curation, data science, and technical interoperability from institutions hosting researchers to those hosting data. It may further depend on the willingness of funders to support the creation and sustainability of data centers and their networks. Although expensive for single data stewards, model-to-data may be more efficient overall by reducing the need for redundant, secure computing environments.

We are cautiously optimistic that model-to-data can unlock at least some data sets for broader sharing, for at least some uses, but we remain skeptical that it can fully square the circle. To successfully adopt an model-to-data approach, the concept of data stewardship needs to be reconfigured, with implications that reach the organization’s core business model. At least for now, responsible scientific, ethical, and legal data governance remains a reliably human endeavor.

## Abbreviations

API: application programming interface
FAIR: findable, accessible, interoperable, reusable
GDPR: General Data Protection Regulation

## References

[1] National Academy of Science, Engineering, and Medicine (2018). Open Science by Design: Realizing a Vision for 21st Century Research.

[2] Research Funders' Open Access Policies. Sherpa Services.

[3] (2015). The HIPAA Privacy Rule. The US Department of Health and Human Services (HHS).

[4] (2017). Data Protection in the EU. European Commission.

[5] Secondary Use of Health and Social Data. Finnish Ministry of Social Affairs And Health.

[6] International Compilation of Human Research Standards. The US Department of Health and Human Services (HHS).

[7] Kim J (2019). Overview of disciplinary data sharing practices and promotion of open data in science. Sci Educ.

[8] Council of Canadian Academies.

[9] Stein LD, Knoppers BM, Campbell P, Getz G, Korbel JO (2015). Data analysis: create a cloud commons. Nature.

[10] Burton PR, Murtagh MJ, Boyd A, Williams JB, Dove ES, Wallace SE, Tassé AM, Little J, Chisholm RL, Gaye A, Hveem K, Brookes AJ, Goodwin P, Fistein J, Bobrow M, Knoppers BM (2015). Data safe havens in health research and healthcare. Bioinformatics.

[11] Platt R, Lieu T (2018). Data enclaves for sharing information derived from clinical and administrative data. J Am Med Assoc.

[12] CMS Virtual Research Data Center (VRDC). ResDAC.

[13] Guinney J, Saez-Rodriguez J (2018). Alternative models for sharing confidential biomedical data. Nat Biotechnol.

[14] Ellrott K, Buchanan A, Creason A, Mason M, Schaffter T, Hoff B, Eddy J, Chilton JM, Yu T, Stuart JM, Saez-Rodriguez J, Stolovitzky G, Boutros PC, Guinney J (2019). Reproducible biomedical benchmarking in the cloud: lessons from crowd-sourced data challenges. Genome Biol.

[15] Mason M, Amatangelo M, Auclair D, Bassett D, Dai H, Dervan A (2017). Abstract 4725: multiple myeloma dream challenge: a crowd-sourced challenge to improve identification of high-risk patients. Clin Res.

[16] Austin LM, Lie D (2019). Safe Sharing Sites. NYU Law Review.

[17] #opendata4covid19. Health Insurance Review & Assessment Service.

[18] Schadt EE (2012). The changing privacy landscape in the era of big data. Mol Syst Biol.

[19] All In: Data for Community Health.

[20] Garza M, del Fiol G, Tenenbaum J, Walden A, Zozus MN (2016). Evaluating common data models for use with a longitudinal community registry. J Biomed Inform.

[21] Contreras JL, Reichman JH (2015). Sharing by design: data and decentralized commons. Science.

[22] All of Us. National Institutes of Health.

[23] Federated Data Systems: Balancing Innovation and Trust in the Use of Sensitive Data. The World Economic Forum.

[24] Beyan O, Choudhury A, van Soest J, Kohlbacher O, Zimmermann L, Stenzhorn H, Karim MR, Dumontier M, Decker S, da Silva Santos LO, Dekker A (2020). Distributed analytics on sensitive medical data: the personal health train. Data Intell.

[25] (2019). GA4GH Passports and the Authorization and Authentication Infrastructure. GA4GH.

[26] Aarestrup AK, Væver MS, Petersen J, Røhder K, Schiøtz M (2020). An early intervention to promote maternal sensitivity in the perinatal period for women with psychosocial vulnerabilities: study protocol of a randomized controlled trial. BMC Psychol.

[27] European Translational Information and Knowledge Management Services: eTRIKS: Deliverable Report. eTRIKS.

[28] Kunz (2019). Empowering Distributed Analysis Across Federated Cohort Data Repositories Adhering to FAIR Principles. ERCIM News 121.

[29] Research on European Children and Adults Born Preterm.

[30] EUCAN-Connect.

[31] Wilkinson MD, Dumontier M, Aalbersberg IJ, Appleton G, Axton M, Baak A, Blomberg N, Boiten J, da Silva Santos LB, Bourne PE, Bouwman J, Brookes AJ, Clark T, Crosas M, Dillo I, Dumon O, Edmunds S, Evelo CT, Finkers R, Gonzalez-Beltran A, Gray AJ, Groth P, Goble C, Grethe JS, Heringa J, 't Hoen PA, Hooft R, Kuhn T, Kok R, Kok J, Lusher SJ, Martone ME, Mons A, Packer AL, Persson B, Rocca-Serra P, Roos M, van Schaik R, Sansone S, Schultes E, Sengstag T, Slater T, Strawn G, Swertz MA, Thompson M, van der Lei J, van Mulligen E, Velterop J, Waagmeester A, Wittenburg P, Wolstencroft K, Zhao J, Mons B (2016). The FAIR guiding principles for scientific data management and stewardship. Sci Data.

[32] Fiume M, Cupak M, Keenan S, Rambla J, de la Torre S, Dyke SO, Brookes AJ, Carey K, Lloyd D, Goodhand P, Haeussler M, Baudis M, Stockinger H, Dolman L, Lappalainen I, Törnroos J, Linden M, Spalding JD, Ur-Rehman S, Page A, Flicek P, Sherry S, Haussler D, Varma S, Saunders G, Scollen S (2019). Federated discovery and sharing of genomic data using beacons. Nat Biotechnol.

[33] The Global Alliance for Genomics and Health (GA4GH).

[34] Global Alliance for Genomics and Health (2016). GENOMICS. A federated ecosystem for sharing genomic, clinical data. Science.

[35] GA4GH/Wiki. GitHub.

[36] The Next Generation Cancer Knowledge Network. NCI Genomic Data Commons - National Cancer Institute.

[37] CanDIG.

[38] Patil D, Mason H (2015). Data Driven.

[39] Schreier J, Feeney R, Keeling P (2019). Diagnostics reform and harmonization of clinical laboratory testing. J Mol Diagn.

[40] Rocher L, Hendrickx JM, de Montjoye Y (2019). Estimating the success of re-identifications in incomplete datasets using generative models. Nat Commun.

[41] Regulatory & Ethics Toolkit. Global Alliance for Genomics and Health.

[42] Carlini N, Liu C, Erlingsson U, Kos J, Song D (2018). The Secret Sharer: Evaluating and Testing Unintended Memorization in Neural Networks.

[43] Paltoo DN, Rodriguez LL, Feolo M, Gillanders E, Ramos EM, Rutter JL, Sherry S, Wang VO, Bailey A, Baker R, Caulder M, Harris EL, Langlais K, Leeds H, Luetkemeier E, Paine T, Roomian T, Tryka K, Patterson A, Green ED, National Institutes of Health Genomic Data Sharing Governance Committees (2014). Data use under the NIH GWAS data sharing policy and future directions. Nat Genet.

[44] Townend D, Dove ES, Nicol D, Bovenberg J, Knoppers BM (2016). Streamlining ethical review of data intensive research. Br Med J.

[45] Baker DB, Knoppers BM, Phillips M, van Enckevort D, Kaufmann P, Lochmuller H, Taruscio D (2019). Privacy-preserving linkage of genomic and clinical data sets. IEEE/ACM Trans Comput Biol Bioinform.

[46] Oprisanu B, de Cristofaro E (2018). AnoniMME: bringing anonymity to the matchmaker exchange platform for rare disease gene discovery. Bioinformatics.

[47] Lamprecht A, Garcia L, Kuzak M, Martinez C, Arcila R, del Pico E, del Angel VD, van de Sandt S, Ison J, Martinez PA, McQuilton P, Valencia A, Harrow J, Psomopoulos F, Gelpi JL, Chue Hong N, Goble C, Capella-Gutierrez S (2020). Towards FAIR principles for research software. DS.

[48] Birney E, Hudson TJ, Green ED, Gunter C, Eddy S, Rogers J, Harris JR, Ehrlich SD, Apweiler R, Austin CP, Berglund L, Bobrow M, Bountra C, Brookes AJ, Cambon-Thomsen A, Carter NP, Chisholm RL, Contreras JL, Cooke RM, Crosby WL, Dewar K, Durbin R, Dyke SO, Ecker JR, El Emam K, Feuk L, Gabriel SB, Gallacher J, Gelbart WM, Granell A, Guarner F, Hubbard T, Jackson SA, Jennings JL, Joly Y, Jones SM, Kaye J, Kennedy KL, Knoppers BM, Kyrpides NC, Lowrance WW, Luo J, MacKay JJ, Martín-Rivera L, McCombie WR, McPherson JD, Miller L, Miller W, Moerman D, Mooser V, Morton CC, Ostell JM, Ouellette BF, Parkhill J, Raina Ps, Rawlings C, Scherer SE, Scherer SW, Schofield PN, Sensen CW, Stodden VC, Sussman MR, Tanaka T, Thornton J, Tsunoda T, Valle D, Vuorio EI, Walker NM, Wallace S, Weinstock G, Whitman WB, Worley KC, Wu C, Wu J, Yu J, Toronto International Data Release Workshop Authors (2009). Prepublication data sharing. Nature.

[49] Longo DL, Drazen JM (2016). Data sharing. N Engl J Med.

[50] Anderson W, Apweiler R, Bateman A, Bauer G, Berman H, Blake J (2017). Towards Coordinated International Support of Core Data Resources for the Life Sciences. bioRxiv.

[51] Dana G, Arnaud B Global Data Access for Solving Rare Disease: A Health Economics Value Framework. The World Economic Forum.

